# Cost-effectiveness analysis of treatment sequences containing tofacitinib for the treatment of rheumatoid arthritis in Spain

**DOI:** 10.1007/s10067-020-05087-3

**Published:** 2020-04-17

**Authors:** F. Navarro, J. M. Martinez-Sesmero, A. Balsa, C. Peral, M. Montoro, M. Valderrama, S. Gómez, F. de Andrés-Nogales, M. A. Casado, Itziar Oyagüez

**Affiliations:** 1 Rheumatology Deparment Hospital Quirón Salud Infanta Luisa, Seville, Spain; 2 Hospital Pharmacy, Hospital Universitario Clínico San Carlos, Madrid, Spain; 3 Rheumatology Department Hospital Universitario La Paz, Madrid, Spain; 4 Pfizer S.L.U, Alcobendas, Madrid Spain; 5 Pharmacoeconomics & Outcomes Research Iberia (PORIB), Paseo Joaquín Rodrigo, 4 letra I, 28224 Pozuelo de Alarcón, Madrid Spain

**Keywords:** Cost-effectiveness, Rheumatoid arthritis, Tofacitinib

## Abstract

**Objective:**

To assess the cost-effectiveness of tofacitinib-containing treatment sequences versus sequences containing only standard biological therapies in patients with moderate-to-severe rheumatoid arthritis (RA) after the failure of conventional synthetic disease-modifying antirheumatic drugs (csDMARD-IR population) and in patients previously treated with methotrexate (MTX) who show an inadequate response to second-line therapy with any tumour necrosis factor inhibitor (TNFi-IR population).

**Methods:**

A patient-level microsimulation model estimated, from the perspective of the Spanish Public NHS, lifetime costs and quality-adjusted life years (QALY) for treatment sequences starting with tofacitinib (5 mg twice daily) followed by biological therapies versus sequences of biological treatments only. Concomitant treatment with MTX was considered. Model’s parameters comprised demographic and clinical inputs (initial Health Assessment Questionnaire [HAQ] score and clinical response to short- and long-term treatment). Efficacy was measured by means of HAQ score changes using mixed treatment comparisons and data from long-term extension (LTE) trials. Serious adverse events (SAEs) data were derived from the literature. Total cost estimation (€, 2018) included drug acquisition, parenteral administration, disease progression and SAE management.

**Results:**

In the csDMARD-IR population, sequences starting with tofacitinib proved dominant options (more QALYs and lower costs) versus the corresponding sequences without tofacitinib. In the TNFi-IR population, first-line treatment with tofacitinib+MTX followed by scAbatacept+MTX➔rituximab+MTX➔certolizumab+MTX proved dominant versus scTocilizumab+MTX➔scAbatacept+MTX➔rituximab+MTX➔certolizumab+MTX; and tofacitinib+MTX➔scTocilizumab+MTX➔scAbatacept+MTX➔rituximab+MTX versus scTocilizumab+MTX➔scAbatacept+MTX➔rituximab+MTX➔certolizumab+MTX was less effective but remained a cost-saving option.

**Conclusions:**

Inclusion of tofacitinib seems a dominant strategy in moderate-to-severe RA patients after csDMARDs failure. Tofacitinib, as initial third-line therapy, proved a cost-saving strategy (€− 337,489/QALY foregone) in moderate-to-severe TNFi-IR RA patients.

**Table:** 

**Key points** *• Therapeutical approach in rheumatoid arthritis (RA) consisted in sequences of several therapies during patient lifetime.* *• Treatment sequences initiating with tofacitinib followed by biological drugs provided higher health effects in csDMARDs-IR population, compared with sequences containing only biological drugs.* *• In both csDMARD-IR and TNFi-IR RA populations, initiating treatment with tofacitinib was associated to lower treatment costs for the Spanish National Health System.*

## Introduction

Rheumatoid arthritis (RA) is a chronic and progressive autoimmune disease associated with long-term morbidity, increased mortality [1] and a decline in patients’ quality of life [2].

A recent study showed that the prevalence of RA in Spain is to 0.9% [3]. RA generates a significant cost for the Spanish National Health System (NHS). In 2001, RA-derived costs exceeded 2.25 billion euros, with healthcare costs, mainly associated to the disease-derived disability, representing 70% of the total [4].

RA treatment guidelines recommend methotrexate (MTX) or other synthetic disease-modifying antirheumatic drugs (csDMARDs) as the first therapeutic option. If the therapeutic objective is not achieved with csDMARDs, then start treatment with advanced therapies, as biological DMARDs (bDMARDs) or targeted synthetic DMARDs (tsDMARDs) is recommended [5].

The bDMARDs was a relevant fact in the management of RA in the past decades. Nevertheless, some patients still show an inadequate response to treatment with bDMARDs and do not achieve an adequate control of the disease [5, 6].

Janus kinase (JAK) inhibitors, currently classified as tsDMARDs, constitute a new alternative in the treatment of immune-mediated diseases and RA in particular [7]. In addition to a new mechanism of action, oral administration brings advantages such as ease and convenient administration, being an interesting alternative to the parenteral route of bDMARDs [8].

The European League Against Rheumatism (EULAR) guidelines recommend the use of tsDMARDs or bDMARDs, preferably in combination with MTX, in patients with poor prognostic factors who present an inadequate response to previous first-line treatment with csDMARDs. Monotherapy is reserved for patients for whom csDMARDs are contraindicated, with tsDMARDs and IL-6 receptor antagonists being preferred over other options. In patients who do not respond to bDMARDs or tsDMARDs, the administration of another drug from these groups is recommended [5].

The use of tofacitinib in RA was approved by the European Medicines Agency (EMA) in March 2017 and by the US Food and Drug Administration (FDA) in November 2012 and includes its combined administration with MTX for the treatment of active moderate-to-severe RA in adult patients who have not adequately responded or are intolerant to one or several csDMARDs, as well as its use in monotherapy in case of intolerance to MTX or when treatment with MTX proves inadequate [9]. The use of baricitinib was approved by EMA in February 2017 (4 and 2 mg) for the treatment of active moderate-to-severe RA in adult patients with an inadequate response or intolerance to one or several DMARDs, with the option for use in monotherapy or in combination with MTX, and by the FDA in May 2018 (2 mg) for the treatment of active moderate-to-severe RA in adult patients with an inadequate response to one or several bDMARDs [10].

The objective of this study was to assess the efficiency of positioning tofacitinib as initial therapy of the treatment sequence in patients with moderate-to-severe RA, in two types of subpopulations; csDMARDs-IR population (patients with an inadequate response or intolerance to previous therapy with csDMARD) and TNFi-IR population (patients previously treated with anti-TNF who did not achieve an adequate response or who are intolerant to said therapy). The objective is therefore to provide information that supports the decision-making process with regard to selecting a RA treatment sequence in the two subpopulations analysed.

## Materials and methods

### Economic model

The efficiency of the treatment sequences containing tofacitinib was calculated by adapting a cost-effectiveness analysis model, previously used in other countries’ healthcare settings [11–13], to the Spanish context. Main characteristics of this model have been published [11, 12]. Its consists of a microsimulation, the structure of which is based on models used previously to assess the efficiency of other bDMARs in RA [14–16]. The patient-level simulation is a widely-used technique for RA modelling due to the clinical management characteristics, duration and use of sequential treatments. Unlike traditional Markov models which analyse cohorts of patients, in microsimulation’ models individual patients move through the different stages of the model one-by-one during the simulation. This allows time to be calculated in each treatment line and the associated costs to be applied to the first treatment cycle (such as induction therapy or patient education costs). Moreover, it also allows a patient history to be compiled to determine future events, time on treatment, costs and quality of life.

Our analysis included 100,000 patient-level simulation iterations, based on which the average costs and health outcomes of the treatment sequences assessed were obtained. The Incremental cost-effectiveness ratio (ICER) was determined among the treatment sequences assessed through an incremental comparison of costs and outcomes, applying an annual discount rate of 3% [17].

**Fig. 1:**
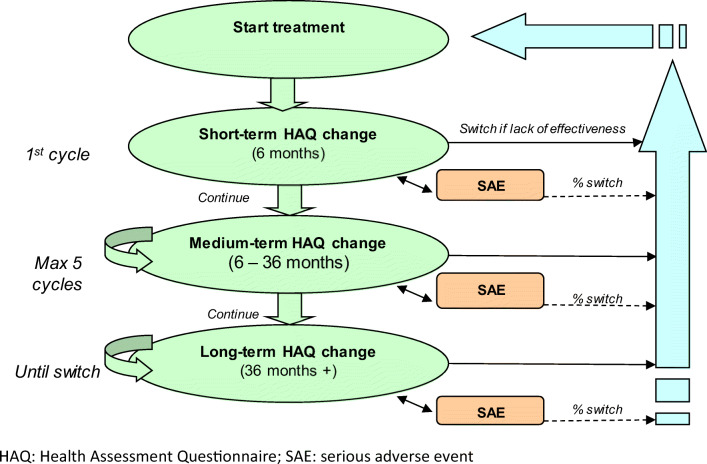
Diagram of the model. HAQ Health Assessment Questionnaire, SAE serious adverse event

The analysis period covered each patient’s lifetime, whose pathway through the model was assessed in six-month cycles (Fig. 1). This period was deemed to be the most suitable interval for performing follow-up that allowed to detect changes in the disease’s course and to compile the resources consumption, as reported monitoring frequency in clinical trials and routine clinical practice assessment [18].

Disease progression was determined using the Health Assessment Questionnaire (HAQ) score. Mortality and quality of life are related to the HAQ score, rather than being associated with a specific state of health.

During the simulation, patients are allowed to stop their current treatment and move to next treatment line established in the sequence within the first six months of treatment (based on HAQ score variation), due to subsequent loss of response (> 6 months of treatment) or following the onset of serious adverse events (SAEs).

Patient’s HAQ score and their score-adjusted likelihood of suffering a SAE, are determined in each six-month cycle, conditioning the continuity of the therapy and ensuring that patients who remained on treatment are those benefitting from therapy. If a patient’s HAQ score changes enough to be considered an inadequate initial response or a loss of treatment response, the patient moves on to the next treatment line and returns to the start of the model diagram with the new treatment and an initial HAQ score equivalent to the pre-treatment score, thereby preventing patients with the greatest frequency of treatment changes from presenting accumulated benefits.

Treatment response was set to be assessed at three different intervals: short-term (first 6 months), medium-term (6–36 months) and long-term (> 36 months of treatment). The variation in HAQ score required for switching varies according to time. Treatment response is more pronounced in the first six months and LTE studies have demonstrated that improvements in HAQ become less sensitive to therapy over time (Fig. 1).

After starting a new therapy, to consider an adequate response at month six the improvement in the HAQ score has to be at least 0.35, and subsequently any decrease in the HAQ score would mean changes of less than 0.35 every six months. These thresholds were estimated by using a linear regression function for conversion of a 1.2 point improvement in the DAS28 (Disease Activity Score) to HAQ scores. Data from a trial comparing etanercept and MTX (TEMPO trial) [19] were used to establish the relationship between DAS28 and HAQ, based upon the expert opinion which stated that the minimum improvement in DAS28 required when a patient initially starts a new therapy is 1.2.

### Population

The study included RA patients who had active moderate-to-severe RA and who were eligible for treatment with tofacitinib, according to the authorized indication [9]. In one group, RA patients DMARDs-IR, while the other one included RA patients TNFi-IR population [20].

The profile of both populations (Table 1) was defined by the age and duration of RA of patients in the Biobadaser database [22]. Moreover, the average weight of the Spanish population aged over 45 [23] and the initial HAQ score of the DMARDs-IR population in clinical trials with tofacitinib were considered. These were similar to HAQ value in the Spanish CREATE registry [21] (patients with inflammatory rheumatic disease starting treatment with bDMARDs) based on the initial DAS28 score (HAQ = − 0.185 + (0.289 × DAS28)) (Table 1).

**Table 1:** Patients’ baseline characteristics

	Patient population	
	csDMARD-IR	TNFi-IR
Initial HAQ score	1.45 [21]	
Age in years, mean (SD)	47.7 (16.3) [22]	51.0 (14.2) [22]
Gender (% male)	39.6% [22]	34.9% [22]
Mean weight (kg)	72.02 (> 45 years) [23]	72.02 (> 45 years) [23]
RA duration at start of simulation (years)	6.2 [95% CI 2.2–12.8] [22]	10.6 [95% CI 5.7–17.6] [22]

*CI*, confidence interval; *csDMARD-IR*, patients with an inadequate response or intolerance to previous therapy with a conventional synthetic disease-modifying antirheumatic drug; *HAQ*, Health Assessment Questionnaire; *RA*, rheumatoid arthritis; *SD*, standard deviation; *TNFi-IR*, patients with an inadequate response or intolerance to previous therapy with a tumour necrosis factor inhibitor

This model did not compare two therapies individually, but it assessed therapeutic sequences with up to five possible treatment lines. All the therapies in all the sequences include combination with MTX.

The analyses included two scenarios for the DMARDs-IR population, and two scenarios for the TNFi-IR population, where the patients had received MTX in first-line therapy and a TNF inhibitor in the second line, before starting the sequences assessed in the analysis.

**Fig. 2:**
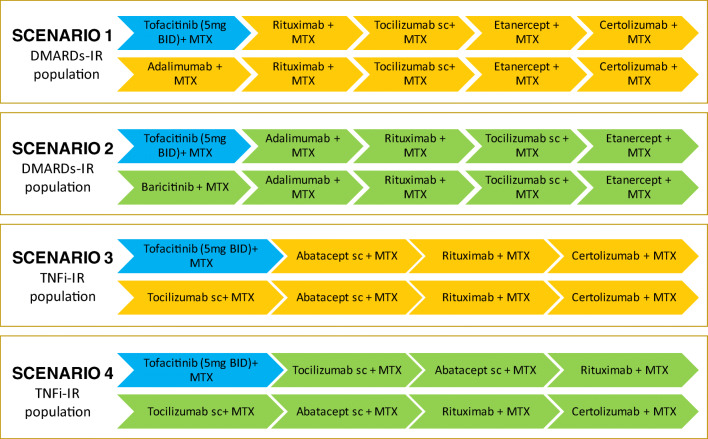
Scenarios compared. BID, twice daily; csDMARD-IR, patients with an inadequate response or intolerance to previous therapy with a disease-modifying antirheumatic drug; MTX, methotrexate; sc, subcutaneous; TNFi-IR, patients with an inadequate response or intolerance to previous therapy with a tumour necrosis factor inhibitor

Among the wide variety of combinations of therapies, the preferred sequences as most likely to be used in clinical practice were defined by the expert panel (Fig. 2).

These sequences were compared with an alternative sequence after replacing by tofacitinib (5 mg twice daily [BID]) the initial therapy of the sequences, in order to assess, the efficiency of positioning tofacitinib at initial therapy of the treatment sequence.

For TNFi-IR population, sequence was selected to include mechanism of actions others than anti-TNFs. The efficiency of tofacitinib was assessed by replacing subcutaneous tocilizumab by tofacitinib or by positioning tofacitinib before the administration of tocilizumab.

The active sequences including tofacitinib as initial line therapy were compared with the comparator sequences containing other bDMARDs. Dosages for these bDMARDs derived from the correspondent Summaries of Product Characteristic.

As it was done in previous evaluations with the global model [11, 12], the clinical efficacy data, in terms of HAQ score, for the initial treatment response, were obtained from a meta-analysis derived from a systematic literature review of studies on the efficacy of different treatments for moderate-to-severe RA [24, 25]. For therapies in which no efficacy data were found in terms of the HAQ score, efficacy was determined using the ACR values obtained from the literature, by means of the conversion proposed in National Institute for Health and Care Excellence (NICE) Technology Appraisal 198 [26].

Limited data about HAQ change in TNFi-IR population were identified in the systematic literature for the network meta-analysis [24]. When available the effectiveness data on the TNFi-IR population were applied to the population of patients who had changed treatment from a TNFi. Otherwise, we adopted the information available on csDMARD-IR patients.

Medium-term responses were obtained from the literature [27–33] and there was assumed to be no long-term progression in patients receiving treatment with DMARDs (Table 2).

**Table 2:** Changes in HAQ scores by therapy and response period

Mean change in HAQ score (SD) per half-yearly period									
Period		ABA	ADA	BAR	CTZ	ETN	RTX	TCZ	TOF
Short term (first 6 months of treatment)	csDMARD-IR population	− 0.790 (0.408) [24]	− 0.637 (0.350) [25]	− 0.564^a^ (0.512) [25]	− 0.764 (0.390) [25]	− 0.377 (0.486) [25]	− 0.813 (0.379) [25]	− 0.562 (0.351) [25]	− 0.564 (0.512) [25]
	TNFi-IR population	− 0.360	− 0.409	− 0.447^a^	− 0.350	− 0.393	− 0.407	− 0.437	− 0.447
Medium term (6–36 months of treatment)		− 0.050 (0.25^b^) [27]	− 0.030 (0.25^b^) [28]	− 0.016 (0.00573^a^)	− 0.05 (0.25^b^)	0 (0.25^b^) [29]	− 0.010 (0.25^b^) [31]	− 0.0126 (0.25^b^) [33]	− 0.016 (0.00573) [32]
Long term (> 36 months of treatment)		0 (0.15^b^)	0 (0.15^b^)	0 (0.15^b^)	0 (0.15^b^)	0 (0.15^b^)	0 (0.15^b^)	0 (0.15^b^)	0 (0.15^b^)
Safety [36]									
SAE proportion per 6 months		1.509%	2.489%	1.499%^a^	3.724%	2.010%	1.843%	2.688%	1.499%

^a^Assumption. Equivalence to tofacitinib, ^b^assumption for SD

*ABA*, abatacept; *ADA*, adalimumab; *BAR*, baricitinib; *CTZ*, certolizumab; *csDMARD-IR*, patients with an inadequate response or intolerance to previous therapy with a disease-modifying antirheumatic drug; *ETN*, etanercept; *HAQ*, Health Assessment Questionnaire; *RTX*, rituximab; *SD*, standard deviation; *TCZ*, tocilizumab; *TNFi-IR*, patients with an inadequate response or intolerance to previous therapy with a tumour necrosis factor inhibitor; *TOF*, tofacitinib

### Adverse events

The model assesses the safety profile of tofacitinib in comparison to bDMARDs. Given the heterogeneity of their SAEs reported, we opted to model them all together, considering the onset of severe infections (SI) as being representative of the most common SAEs in RA patients, and applying the frequencies published [35], in line with the approaches previously done [11, 34].

The proportion of patients that switched to the following treatment on the defined sequence if an SAE occurred was established at 74%, for all the therapies included, based on the analysis of individualized data from the studies identified in the indirect comparison carried out [24], and in the same way that previous studies with the global model [12].

### Mortality

The functional status of the patients and RA severity are directly related to increased mortality in RA [36] so additional benefits will be associated to disease progression reduction, resulting from a reduction in mortality.

In this microsimulation model [11], mortality rate by age and gender due to any cause in the Spanish population was obtained from the Spanish National Institute of Statistics [23] and it was adjusted based on the corresponding HAQ value, using the ratio described in previous publications [37].

### Quality of life

The effect on health-related quality of life was considered in the model by estimating the quality-adjusted life years (QALYs). The correlation between the HAQ score and utilities was estimated [38] (Table 2), moreover and similarly to previous modellings [11] over a four-week period, which would equate to a QALY loss of 0.012 in patients with SAEs.

### Costs

Our analysis was developed from the perspective of the Spanish NHS. Due to lack of accurate evidence, the societal perspective was not assessed. The direct healthcare costs included were: drug-acquisition costs, administration costs, disease management costs according to severity and SAE costs.

Acquisition costs were estimated using the ex-factory price (EFP) [39] including the corresponding mandatory deduction outlined in Royal Decree 08/2010, the reference price established or the lowest EFP among the available biosimilars. These costs were calculated for two different periods: cost in the first six months of treatment (including induction dose where appropriate) and half-yearly cost from the sixth month of treatment, considering the dosages established in the summaries of product characteristics which are shown in Table 3. In the case of rituximab, cycles of two infusions are recommended, with the need for retreatment assessed 24 weeks after the last cycle. To reflect clinical practice in Spain, the administration of further rituximab cycles was considered every nine months [41] by weighting the cost to the six-month cycle period.

**Table 3:** Unit costs (€, 2018)

Treatment acquisition			Unit cost (€, 2018)	
Therapy	No. of administrations in 6 months		EFP [39] with Royal Decree 08/2010 deduction or reference price	Pharmacological cost of the therapy in combination with MTX, per 6-month period
	Initial period	Subsequent periods		
Abatacept 125 mg	26	26	€194.42	€5055.06
Adalimumab 40 mg	13	13	€404.25**	€5255.37
Baricitinib 4 mg	182	182	€31.08	€5672.24
Certolizumab 200 mg	13	13	€438.45	€5699.99
Etanercept 50 mg	26	26	€169.07*	€4395.96
Rituximab 500 mg	1.33	1.33	€970.65**	€1294.34
Tocilizumab 162 mg	26	26	€225.98	€5875.62
Tofacitinib 5 mg	365	365	€13.61	€4968.05
Methotrexate 2.5 mg	26	26	€0.05	-
Healthcare resources			Unit cost (€, 2018)	
Training session for subcutaneous self-administration			€27.10 [40]	
Infusion at day hospital			€252.42 [40]	
Initial rheumatologist consultation			€111.52 [40]	
Subsequent rheumatologist consultation			€66.91 [40]	
Severe infection management			€5871.08 [40]	

*EFP*, ex-factory price; *MTX*, methotrexate

*Reference price

**Lowest price of the biosimilar products available on Spanish market

Administration-related costs included the cost of parenteral administration at the day hospital for intravenous drugs, or the cost of patient’s education in self-administration of subcutaneous medicines. The unit costs of these resources were obtained from eSalud [40], a Spanish database covering healthcare costs in the country.

The costs of managing RA according to severity were estimated using the published evidence [4]. The costs of managing a SAE were equated to managing a SI, and it was derived from the average costs per diagnosis-related group and autonomous community fees for the types of infection selected which, in line with the available evidence [42] and after validation by a panel of clinical experts, included these SI (without complications): septicaemia, acute bronchitis, pneumonia, pulmonary tuberculosis, kidney and urinary tract infections and respiratory inflammation.

All costs were expressed in euros (2018 values) (Table 3).

### Sensitivity analysis

Considering the stochastic feature of the microsimulation models, a probabilistic sensitivity analysis (PSA) were carried out with the implementation of two nested loops. An inner loop reflecting the first-order uncertainty in which mean cost and mean QALYs are calculated from the 1000 simulated patients sample, by applying a normal distribution to cohort characteristics inputs (age, HAQ initial score, weight and RA duration). The outer loop allows for the second-order variation by randomly varying parameters around the HAQ relationships (utilities, mortality, costs) by randomly drawing the parameter from a normal distribution. The first loop was repeated for different parameters set up to obtain 100 cost-effectiveness estimations.

## Results

This analysis assessed the efficiency of several treatment sequences, consisting of the administration of tofacitinib (5 mg BID) and subsequent lines of bDMARDs in two different populations (csDMARD-IR and TNFi-IR), compared with alternative sequences which did not consider the administration of tofacitinib (Table 4).

**Table 4:** Lifetime results for base case

csDMARD-IR population						
	Scenario 1			Scenario 2		
	Active sequence	Comparator sequence	Difference	Active sequence	Comparator sequence	Difference
QALYs	13.991	13.924	0.067	13.753	13.620	0.133
Total cost	€224,143	€229,926	€− 5783	€225,851	€239,826	€− 13,975
Pharmacological cost	€164,786	€164,802	€− 16	€173,644	€187,969	€− 14,326
Administration cost	€27,928	€30,861	€− 2933	€19,689	€18,609	€1080
Disease management cost	€28,116	€28,513	€− 397	€29,570	€30,526	€− 955
SAE cost	€3313	€5750	€− 2437	€2948	€2722	€226
ICER	Active sequence dominant			Active sequence dominant		
TNFi-IR population						
	Scenario 3			Scenario 4		
	Active sequence	Comparator sequence	Difference	Active sequence	Comparator sequence	Difference
QALYs	14.199	14.064	0.135	13.950	14.042	− 0.092
Total cost	€242,312	€279,426	€− 37,115	€248,194	€279,243	€− 31,049
Pharmacological cost	€180,778	€188,727	€− 7949	€175,816	€188,541	€− 12,725
Administration cost	€31,739	€57,500	€− 25,761	€41,280	€57,401	€− 16,121
Disease management cost	€26,879	€27,754	€− 875	€28,422	€27,833	€590
SAE cost	€2916	€5445	€− 2529	€2677	€5469	€− 2793
ICER	Active sequence dominant			Active sequence less effective, but less costly 3rd quadrant* (€− 31,049/− 0.092 QALYs = 337,489€/QALY)		

*csDMARD-IR*, patients with an inadequate response or intolerance to previous therapy with a disease-modifying antirheumatic drug; *ICER*, incremental cost-effectiveness ratio; *QALYs*, quality-adjusted life year; *SAE*, serious adverse event; *TNFi-IR*, patients with an inadequate response or intolerance to previous therapy with a tumour necrosis factor inhibitor

*In the 3rd quadrant of the cost-effectiveness plane (less effective but less costly therapies versus comparator), the decision rule is complementary to the one applied in the 1st quadrant (more effective and more costly therapies). The alternatives can be considered as cost-effective versus the comparator, if the savings associated to the loose of 1 QALY are above the willingness-to-pay threshold. In this case, considering a €25,000/QALY gained threshold, the active sequence would result in a cost-effective option versus the comparator sequence [47]

In the csDMARD-IR population, the base case scenario (scenario 1, Fig. 2), that compared the sequence with tofacitinib+MTX as first-line therapy versus the alternative with adalimumab biosimilar+MTX as first-line therapy and the same subsequent sequence of other bDMARD, revealed that the sequence with tofacitinib as first-line therapy proved a more effective option.

In the other scenario (scenario 2, Fig. 2), the sequence with tofacitinib would provide 13.75 QALYs compared with the 13.62 QALYs in the alternative sequence.

In both scenarios, the sequences with tofacitinib as first-line therapy were associated with a lower total cost per patient than the comparator sequences, with total cost savings ranging from €− 5783 (scenario 1) to €− 13,975 (scenario 2) for the lifetime period simulated.

For the csDMARD-IR population, the sequences with tofacitinib would prove dominant strategies (more effective and less costly) versus the alternatives in the two scenarios assessed.

In the scenarios analysed for the TNFi-IR population, the sequences starting with tofacitinib were associated with lower total costs than the alternative sequences. The sequence in scenario 3 (Fig. 2) starting with tofacitinib+MTX proved more effective (0.135 additional QALYs) than the comparator, leading it to be classified as the dominant sequence. In the other scenario proposed for the TNFi-IR population (scenario 4, Fig. 2), the sequence starting with tofacitinib provided 0.092 QALYs less than the alternative sequence.

The results of the PSA showed that, of the 10,000 iterations carried out in each case, the sequences with tofacitinib, due to being below the threshold [45] of €25,000/additional QALY with respect to the comparator sequences without tofacitinib, could be considered cost-effective in 64.0%, 56.8%, 59.9% and 76.3% for scenarios 1, 2, 3 and 4, respectively.

## Discussion

There are many publications exploring the cost-effectiveness of individual therapies for RA patients; however, a lack of high-quality evaluations assessing bDMARD sequences has been recently demonstrated [46]. This work assesses the health benefits and total healthcare costs assumed by NHS of different treatment sequences in RA patients in Spain, to determine the efficiency of treatment strategies starting with tofacitinib both in the csDMARD-IR and TNFi-IR populations.

The efficiency of the treatment sequences was determined using a patient-level microsimulation [14, 15]. Analysing sequences, rather than making traditional head-to-head comparisons, is aligned with current recommendations that [5, 33] recommend the sequential use of DMARD until the disease is controlled.

In the analysis, the therapeutic sequences with tofacitinib showed similar health outcomes in all the scenarios assessed. QALYs gained were slightly favourable for three of the four scenarios assessed (two in the csDMARD-IR population and one in the TNFi-IR patients), probably due to the first-order uncertainty around the HAQ scores. In the remaining scenario (scenario 4 in the TNFi-IR population), the treatment sequence starting with tofacitinib proved less effective, but remained the cost savings compared with the comparator sequence. It could be also considered as a cost-effective option if the willingness-to-pay threshold which is used in case of more effective and more costly alternatives (ICER on 1st quadrant of the cost-effectiveness plane) is symmetrical applied for less effective but less costly strategies (ICER on 3rd quadrant of the cost-effectiveness plane) as it is suggested in the literature [47].

The microsimulation model adapted in this work, which allows sequences with up to five treatment lines to be assessed, has previously been used to determine the efficiency of tofacitinib treatment sequences in various countries. In each of them, the sequences with tofacitinib proved more effective, with greater number of QALYs and lower total costs than those without tofacitinib, leading the authors to conclude that the inclusion of tofacitinib would be a dominant option with cost savings for the respective health services. In the single technology appraisal performed by NICE [13] it was concluded that tofacitinib+MTX or as monotherapy could be considered a cost-effective strategy for patients with active severe RA following inadequate response to csDMARD, for whom RTX or MTX are contraindicated or not tolerated.

Other economic evaluation has identified tofacitinib as a cost-effective strategy for the treatment of RA patients in South Korea [48]. Monotherapy with tofacitinib as the second-line treatment after failure with MTX, or in combination with MTX in TNFi-IR patients, proved a less costly option than other biological therapies such as adalimumab, etanercept, certolizumab and tocilizumab [34, 43]. For tofacitinib, other authors estimated a number needed to treat (NNT) and a cost per responder patient similar or lower than that of golimumab, adalimumab and certolizumab, but higher than that of infliximab and etanercept [44]. Administering tofacitinib as a second- or third-line treatment may prove a less costly strategy compared with introducing tofacitinib in the fourth line, following the previous administration of two TNFis after MTX [49].

The main limitation of these results stems from the choice of treatment sequence, since no specific treatments algorithms are outlined in current clinical practice guidelines. The sequences used here seek to reflect the most commonly used regimens in Spanish clinical practice. Facing the lack of published evidence about prescription patterns in Spain, they were defined and validated with the participation of a panel of rheumatologists. Among the wide variety of treatment sequences, the expert panel selected those considered the most likely representative for the vast majority of the RA patients in Spain for both populations. Since the sequences chosen for csDMARD-IR and TNIFi-IR population are different, it is implicitly assumed that the selection of the therapies is independent of the reason of the treatment failure.

The present analysis is focused on the efficiency of positioning tofacitinib as initial therapy of the sequences; however, tofacitinib could be used after the failure of a TNFi in the csDMARD-IR population and the cost-effectiveness of these potential scenarios could be also interesting. Further studies exploring other possibilities would be needed for improvement of the decision-making process.

Since there is a lack of published evidence about the effectiveness for specific treatment sequences, the model was built applying individual efficacy estimates for each therapy independently of the position on the sequence. Equivalent efficacy is applied to one drug, independently of the previous therapy on the sequence, facing the lack of studies about efficacy of drugs following others.

The range of therapies included in the model leads to marked variability in the methodologies and populations included in clinical trials with these drugs. The lack of studies including direct comparisons of sequences or the alternatives assessed forced us to use the results of indirect comparisons obtained from a network meta-analysis [25], which is also one way of minimizing bias associated with the methodological differences between the studies.

The clinical trials used as source for the efficacy of the therapies mostly correspond to international studies. Since populations included in clinical trials are not usually matching with real world patients, some bias could be associated to this issue. However, if such data in the models are representative of local populations has been performed in other economic evaluations, therefore for the present analysis it was considered that they are representative of the Spanish population and healthcare setting. In any case, the use of microsimulation techniques minimizes possible biases as regards the transferability and generalizability of the data to the Spanish population, since each of the simulated patients is defined within the established range for each parameter with the peculiarities of the Spanish population.

Since baricitinib was not included in the network meta-analysis used as main source for the effectiveness, a conservative approach was adopted by assuming equivalence of the health outcomes with tofacitinib. This assumption is justified based on the common mechanism of action of both drugs, and it would be not expected to have great influence on the model results. Further evaluations in the future based on specific data for comparison of tofacitinib and baricitinib could confirm the reliability of the presented results.

Clinical effectiveness in the model is based on HAQ score. Before the lack of data, it was required to map relationships between HAQ and other measurements. In the same way that it was done in other evaluations, a linear regression was used for mapping DAS28 data to HAQ, without it being ruled out that this mapping have provided estimates that are not completely accurate. The paucity of evidence on the long-term effect of the therapies included led equivalent effects to be assumed for each one, in terms of HAQ score progression in the long term, which entails a conservative approach applied previously by other authors [11], with minimal impact on the model results.

In this model mortality is linked to HAQ score, although there is not a global consensus about this approach and other evaluations did not applied the mortality in the same way.

Oral tofacitinib may be associated with reductions in the cost of administering the sequence of up to €− 25,761 per patient with respect to the comparator sequence. In the literature, it is suggested that patients have a certain degree of preference for the oral route over the parenteral route, potentially leading to differences in the outcomes associated with quality of life, but the lack of robust evidence on this aspect prevented it from being included in the model. Thus, by not considering differences in utility values according to the route of administration, the results obtained should be considered conservative since the potential benefit associated with using tofacitinib might be underestimated.

The costs of managing RA were estimated from a study [4] which, although conducted in Spain, may not accurately represent the use of resources currently associated with managing the disease. Therefore, the costs estimated in the model might not coincide with the actual costs incurred by these patients. Given that most of the sequences with tofacitinib were associated with a reduction in the cost of managing the disease, the estimated results might be considered conservative and the use of different costs would not be expected to modify the conclusions reached. Additionally, due to lack of accurate evidence, the societal perspective was not assessed, although the indirect costs pose a great proportion of the RA patients management cost. However, given that disability and their consequences would affect the patients in all the treatment sequences, not big influence on the analysis would be expected. Further investigations developed in the future could provide data to replicate and confirm these results.

Sometimes, the potential statistical differences reported in meta-analyses and/or indirect comparisons between the therapies are not associated with an impact on the clinical field. Based on the available evidence, a cost-effectiveness analysis was chosen instead a cost-minimization analysis; however, the results showed slight differences in the effectiveness of the assessed sequences, suggesting a potential equivalence between them in terms of health outcomes. On the other hand, the sequences initiating with tofacitinib were associated to lower total costs in all the scenarios, so it seems that could be preferred strategies in the management of moderate-to-severe RA patients.

## Conclusions

In conclusion, treatment with tofacitinib (5 mg BID) in combination with MTX in a treatment sequence with antirheumatic drugs provided similar health outcomes that the alternative sequences in the treatment of moderate-to-severe RA provided in intolerant patients or those refractory to previous MTX therapy (csDMARDs-IR population), and in TNFi-IR population. Moreover, sequences with tofacitinib (5 mg BID) resulted cost-saving strategies for the Spanish NHS.

## Data Availability

Additional data are available on request.

## Abbreviations

ACR: American College of Rheumatology
bDMARD: Biological DMARD
BID: Twice daily
csDMARD: Conventional synthetic DMARD
csDMARD-IR: Population intolerant or refractory to previous treatment with csDMARD
DAS: Disease Activity Score
DMARD: Disease-modifying antirheumatic drug
DMARD-IR: Population intolerant or refractory to previous treatment with DMARD
DRG: Diagnosis-related group
EMA: European Medicines Agency
EFP: Ex-factory price
EQ-5D: EuroQol 5 Dimensions questionnaire
EULAR: The European League Against Rheumatism
FDA: US Food and Drug Administration
HAQ: Health Assessment Questionnaire
HUI-3: Health Utilities Index – 3 questionnaire
ICER: Incremental cost-effectiveness ratio
IL-6: Interleukin-6
JAK: Janus Kinase
LTE: Long-term extension
MTX: Methotrexate
NHS: National Health Service
NICE: National Institute for Health and Care Excellence
NNT: Number needed to treat
PSA: Probabilistic sensitivity analysis
QALY: Quality-adjusted life year
RA: Rheumatoid arthritis
SAE: Serious adverse event
SI: Severe infections
TNF: Tumour necrosis factor
TNFi-IR: Population intolerant or refractory to previous treatment with TNF inhibitors
tsDMARD: Targeted synthetic DMARD
